# A Rare Case of Hemophagocytic Lymphohistiocytosis and Macrophage Activation Syndrome in Settings of Etanercept Use and Epstein-Barr Virus Infection

**DOI:** 10.7759/cureus.18507

**Published:** 2021-10-05

**Authors:** Giulio Ciprian, Jessica Khoury, Thomas J Raimondo

**Affiliations:** 1 Internal Medicine, Roger Williams Medical Center, Providence, USA; 2 Critical Care Medicine, Roger Williams Medical Center, Providence, USA

**Keywords:** oncology and critical care, clinical hematology, immune-hematology, auto immune, macrophage activation syndrome (mas), hemophagocytic lymphohistiocytosis (hlh)

## Abstract

Hemophagocytic lymphohistiocytosis (HLH) is a rare syndrome in which widespread activation of the immune system leads to a state of excessive inflammation causing tissue damage. While this condition is mainly established in the pediatric population; due to its rarity, physicians often do not suspect this condition in adults. However, while diagnostic criteria are based on protocols tailored for the pediatric society, recognizing this condition in a timely manner in adults is utterly important to prevent a dismal prognosis. In instances where a concomitant rheumatological disorder is present, the syndrome is referred to as macrophage activation syndrome (MAS). This report describes a case of an adult patient who presented with mucosal bleeding and abdominal pain who was later diagnosed with MAS.

## Introduction

Hemophagocytic lymphohistiocytosis (HLH) is a syndrome characterized by an abnormal immune activation causing excessive inflammation. The primary cell types involved in the pathogenesis of HLH include macrophages, natural killer (NK) cells, and cytotoxic cluster of differentiation 8 (CD8+) lymphocytes (CTLs). Normally, NK cells and CTLs regulate macrophages activation through perforin-dependent proteases-cytotoxic lysis. However, in HLH, this mechanism fails, leading to elevated levels of cytokines and, ultimately, causing widespread tissue damage. Moreover, the activation of macrophages results in the phagocytosis of host blood cells. This phenomenon of engulfment can be observed in biopsies of various immune tissues or bone marrow. However, this is not required to support the diagnosis of HLH. The continuous production of cytokines is confirmed by high levels of chemokines such as chemokine ligand 9 (CXCL9), tumor necrosis factor (TNF)-alpha, IL-6, IL-10, IL-12, and the IL-2 receptor (CD-25), leading to a cytokine storm [1]. If HLH occurs concomitantly with rheumatologic disorders, it is referred to as macrophage activation syndrome (MAS) [2]. A proposed approach to differentiate HLH from MAS is to measure unbound IL-18 levels of >24,000 pg/mL to distinguish the latter from other autoinflammatory conditions [3]. Often, the initial acute episode is triggered by an infection or an alteration in the immune homeostasis. The most common infectious trigger is the Epstein-Barr virus (EBV), which can affect predisposed individuals with a defect in perforin-dependent cytotoxicity, X-linked lymphoproliferative disease (XLP), or even those with sporadic cases. Other causative etiologies include immune checkpoint inhibitors such as nivolumab and ipilimumab. Nonetheless, many immunodeficiency syndromes and genetic mutations have been associated with a role in developing HLH, including familial mutations referred to as familial hemophagocytic lymphohistiocytosis (FHL); however, a more in-depth look goes behind the scope of this case report. Although HLH is primarily a pediatric disease, there have been multiple reports in the adult population as old as 70. The diagnostic criteria adopted for HLH syndrome are based on compatible clinical presentation in the setting of elevated inflammatory markers [ferritin, soluble interleukin-2 receptor (sCD25), and/or CXCL9]. The criteria used in the HLH-2004 trial adopt a more stringent approach, which includes verified HLH-associated mutation positivity, gene defects or other immune regulatory genes, or the following findings: fever >38.5°C; splenomegaly; cytopenia [hemoglobin (Hgb): <9 mg/dL, platelet (Plt): <100,000/mm^3^, absolute neutrophil count: <1000/mm^3^]; hypertriglyceridemia; hemophagocytosis observed on bone marrow, spleen, lymph node, or liver biopsy; low or absent NK cell activity; ferritin >500 ng/mL; and elevated sCD25 or CXCL9 [2]. In this report, we discuss an interesting case of an adult female who presented with abdominal pain, mucosal bleeding, and hypotension who later was found to have HLH/MAS.

## Case presentation

A 60-year-old female with a medical history of rheumatoid arthritis (RA) on weekly injections of etanercept presented to the hospital with weakness, intractable diarrhea, and intermittent abdominal pain associated with nausea and vomiting. On physical examination, she was found to have a petechial rash on the lower extremities, hematuria, and profuse bleeding around the oral mucosa. At the same time, the liver and spleen appeared enlarged on palpation. She was found to have severe acute kidney injury (AKI) [creatinine (Cr): 3.4 mg/dL], electrolytes abnormalities (Na: 129 mEq/L, K: 3.2 mEq/L, Mg: 1.2 mEq/L) and pancytopenia (WBC: 0.3 x10^9^/L, Hgb: 6.7 g/dL, Plt: <1.5 x10^9^/L , absolute neutrophil count: 0.0 x10^9^/L ). Ferritin was recorded above 1,500 ng/mL. She was admitted to ICU with an initial diagnosis of septic shock with unclear source, unresponsive to fluid resuscitation, and therefore started on pressors, along with broad-spectrum antimicrobials (antibiotics, antiviral, and antifungal). The patient received a total of 13 units of platelets without any significant response and eight units of packed red blood cells (pRBCs). Initially, the patient received two doses of filgrastim and intravenous immunoglobulins (IVIGs) due to refractoriness to transfusions, without any significant response. An initial bone marrow biopsy performed to evaluate for pancytopenia was suspicious for lymphoplasmacytic lymphoma (LPL). However, flow cytometry failed to show a clonal population, demonstrating decreased myeloid line and predominance of lymphocytes. Despite the patient spiking occasional fevers, pan-cultures did not yield any pathogen. Immunologic work-up including anti-double-stranded DNA (anti-dsDNA), anti-histidyl tRNA synthetase (anti-Jo-1), anti-scleroderma, anti-Sjögren's syndrome-related antigen A autoantibodies (anti-SS-A), anti-Sjögren's syndrome-related antigen B autoantibodies (anti-SS-B), anti-chromatin antibody (Ab), anti-ribonucleoprotein (anti-RNP), anti-Smith Ab, anti-centromere-B antibodies, and antinuclear antibodies (ANAs) was all negative. Moreover, viral work-up including coxsackievirus, cytomegalovirus (CMV), parvovirus, and human herpesvirus 6 (HHV 6) was negative. However, while anti-cyclic citrullinated peptide was positive as expected in a patient with RA, EBV testing resulted positive (quantitative polymerase chain reaction (qPCR): 113 IU/mL). On the seventh day of hospitalization, a choice of starting romiplostim was made. A day after the infusion, the bone marrow started to show the first signs of recovery, with some immature platelets present in the complete blood count (CBC). While the whole-cell lines showed signs of recovery, the primary team suspected that this condition might be due to hemophagocytic lymphohistiocytosis (HLH) in etanercept use and EBV infection settings. IL-2/CD-25 was elevated at 8,703 pg/mL. After an uneventful recovery, the patient was discharged on a steroid taper initiated as high-dose methylprednisolone (80 mg twice daily) on day 1, with recommendations to follow-up with a hematologist to repeat bone marrow biopsy. The use of etanercept was discouraged, despite literature reporting its use in secondary HLH. Moreover, there are reported cases of bone marrow aplasia while on etanercept therapy; therefore, the safest decision was to terminate it [4-6].

## Discussion

Hemophagocytic lymphohistiocytosis (HLH) is a life-threatening condition. Therefore, it is paramount to successfully diagnose this syndrome early in its course to prompt adequate treatment to achieve complete remission and avoid complications. When a diagnosis of HLH is made, or the clinical suspicion is high, the following steps in the treatment process should be followed to increase the chances of survival. If an active Epstein-Barr virus (EBV) infection is identified, rituximab should be started weekly for four weeks along with intravenous immunoglobulins (IVIGs), which some investigators have recommended. Dexamethasone therapy is recommended from case to case based on the clinical presentation and stability of the patient. If a patient is acutely ill or deteriorating, targeting specific treatment is based on the HLH-94 protocol [6,7]. The treatment consists of eight weeks of induction therapy with etoposide and dexamethasone, with intrathecal addition of hydrocortisone and methotrexate in those with central nervous system (CNS) involvement. Aggressive blood pressure control is vital to decrease the risk of posterior reversible encephalopathy syndrome (PRES), especially in those presenting with headaches, altered mental status, visual disturbances, or seizures. If the patient does not respond to initial therapy, hematopoietic cell transplant (HCT) is considered, as well as the continuation of initial induction therapy past the eight weeks. Some of the indications for HCT include homozygous/compound HLH gene mutations, lack of response to initial therapy, CNS involvement, and presence of hematologic malignancy [8]. Prognosis is poor, with survival approaching 58% in adults and two-month survival in those with an inherited mutation without receiving treatment due to progressive multiorgan failure [9]. A recently developed tool is the HLH-probability calculator that has been created to estimate the risk of having hemophagocytic syndrome (HS) with a score of >250, conferring a 99% probability of HLH [10]. The HScore helps diagnose HLH since it was developed retrospectively, taking into consideration adult patients [7]. The criteria included are similar to the HLH-2004 trial; however, it assigns a score to different variables, as shown in Table 1 [10].

**Table 1:** HScore: Parameters and points assigned for calculation *Suppressed lineage defined as hemoglobin <9.2 g/L, leukocyte count <5 x 10^9^/L, and platelet count <110 x 10^9^/L.

Parameter	Points assigned for each criteria
Known underlying immunosuppression	0 (no) or 18 (yes)
Recorded temperature °C	0 (<38.4), 33 (38.4–39.4), or 49 (>39.4)
Organomegaly (hepatomegaly, splenomegaly)	0 (no), 23 (hepatomegaly or splenomegaly), or 38 (hepatomegaly and splenomegaly)
No. of suppressed bone marrow lineages*	0 (1 lineage), 24 (2 lineages), or 34 (3 lineages)
Ferritin (ug/L)	0 (<2,000), 35 (2,000-6,000), or 50 (>6,000)
Triglycerides (mmol/L)	0 (<1.5), 44 (1.5-4), or 64 (>4)
Fibrinogen (g/L)	0 (>2.5) or 30 (<2.5)
Aspartate aminotransferase levels (U/L)	0 (<30) or 19 (>30)
Hemophagocytosis observed on bone marrow aspirate	0 (no) or 35 (yes)

## Conclusions

Adults are more likely to have a secondary cause of HLH than children and treatment should not be postponed pending testing results when clinical suspicion of HLH is high and when the inclusion criteria are too stringent. HLH can present with other findings such as encephalitis; severe hypotension requiring pressor; renal dysfunction; skin rashes such as purpura, petechiae, and erythroderma; and bleeding with or without underlying associated malignancy. Many of these features are shared among the pediatric and adult population; it is therefore paramount to closely monitor routine laboratory results as well as vital signs and overall clinical picture in order to avoid misdiagnosis.

## References

[1] Humblet-Baron S, Franckaert D, Dooley J (2019). IFN-γ and CD25 drive distinct pathologic features during hemophagocytic lymphohistiocytosis. J Allergy Clin Immunol.

[2] Panagopoulos P, Katsifis G (2018). Secondary hemophagocytic lymphohistiocytosis in a patient with rheumatoid arthritis and vasculitis: a case report and review of the literature. Mediterr J Rheumatol.

[3] Weiss ES, Girard-Guyonvarc'h C, Holzinger D (2018). Interleukin-18 diagnostically distinguishes and pathogenically promotes human and murine macrophage activation syndrome. Blood.

[4] Güven G, Güler A, Özyüncü N, Talan L, Heper A, Turgay TM, Altıntaş ND (2018). Hemophagocytic lymphohistiocytosis after certolizumab treatment in a patient with rheumatoid arthritis. Eur J Rheumatol.

[5] Kozak N, Friedman J, Schattner A (2014). Etanercept-associated transient bone marrow aplasia: a review of the literature and pathogenetic mechanisms. Drugs R D.

[6] Jordan MB, Allen CE, Weitzman S, Filipovich AH, McClain KL (2011). How I treat hemophagocytic lymphohistiocytosis. Blood.

[7] La Rosée P, Horne A, Hines M (2019). Recommendations for the management of hemophagocytic lymphohistiocytosis in adults. Blood.

[8] Ponnatt TS, Lilley CM, Mirza KM (2021). Hemophagocytic lymphohistiocytosis [PREPRINT]. Arch Pathol Lab Med.

[9] Rivière S, Galicier L, Coppo P, Marzac C, Aumont C, Lambotte O, Fardet L (2014). Reactive hemophagocytic syndrome in adults: a retrospective analysis of 162 patients. Am J Med.

[10] Fardet L, Galicier L, Lambotte O (2014). Development and validation of the HScore, a score for the diagnosis of reactive hemophagocytic syndrome. Arthritis Rheumatol.

